# Online physiotherapy for people with axial spondyloarthritis: quantitative and qualitative data from a cohort study

**DOI:** 10.1007/s00296-023-05456-6

**Published:** 2023-09-21

**Authors:** L. Paul, M. T. McDonald, A. McConnachie, S. Siebert, E. H. Coulter

**Affiliations:** 1 School of Health and Life Sciences, Glasgow Caledonian University, Glasgow, G4 0BA UK; 2 Robertson Centre for Biostatistics, School of Health and Wellbeing, University of Glasgow, Glasgow, UK; 3 School of Infection and Immunity, University of Glasgow, Glasgow, UK

**Keywords:** Axial spondyloarthritis, Exercise, Adherence, Internet, Telemedicine

## Abstract

Life-long exercise is essential in axial spondyloarthritis (axSpA) management; however, long-term adherence is challenging. Online exercise programmes are an alternative to face-to-face physiotherapy. (1) To measure adherence to a 12-month, individualised, online physiotherapy programme for people with axSpA, and investigate the effects on disease activity, spinal mobility, work ability, quality of life and function. (2) To investigate associations between programme adherence and outcomes. (3) To explore participants’ views of the programme and factors affecting adherence. Participants were ‘non-exercisers’ recruited from rheumatology outpatient services. Adherence was measured using online diary entries. Outcomes included the BATH indices, health status (EQ5D), Ankylosing Spondylitis Quality of Life (ASQOL), exercise capacity (6MWT), Work, Productivity and Activity Impairment in AS (WPAI), Exercise Attitude Questionnaire (EAQ) and Exercise Motivations Inventory-2 (EMI-2) at baseline, 6 and 12 months. Interviews determined views on the intervention and factors affecting adherence. Fifty participants were recruited. Over the 52-week intervention, adherence (five times/week) ranged from 19% (± 30%) to 44% (± 35%). Significant improvements were found in disease activity (BASDAI), spinal mobility (BASMI), 6MWT, AsQoL and EQ5D-VAS at 6 and 12 months. There were no associations between adherence and baseline variables or demographics. Interviews suggested support from others, routine, and feeling the benefit positively affected adherence. Conversely, lack of motivation, life events and symptoms negatively affected adherence. A 12-month online physiotherapy programme significantly improved symptoms in people with axSpA who were not regular exercisers. Adherence reduced over the intervention period. Online exercise programmes may benefit people with axSpA; however, strategies to improve adherence are required.

**Supplementary Information:**

The online version contains supplementary material available at 10.1007/s00296-023-05456-6.

## Introduction

Axial spondyloarthritis (axSpA) is a chronic inflammatory arthritis which predominantly affects the spine and/or sacroiliac joints. In the UK, the prevalence of axSpA is estimated to be between 0.15 and 1.2%, with symptoms starting from around the second decade [1]. People with axSpA commonly suffer from pain, spinal stiffness, fatigue and functional limitations when performing daily tasks and the condition is associated with an increase in all-cause mortality compared with the general population, predominantly related to osteoporotic fractures and cardiovascular disease [2, 3].

Although exercise is recommended as part of treatment guidelines [4], the optimal long-term delivery of exercise for people with axSpA is unclear. A Cochrane review reported that both home and supervised exercises were beneficial for people with ankylosing spondylitis (AS) but concluded that supervised exercise programmes were more effective than home exercise [5]. The chronic nature of axSpA requires regular on-going exercise, making reliance on supervised programmes prohibitive due to cost and the required commitment from people with axSpA. Previous literature highlights variations in the mode of delivery of exercise programmes (supervised, home, group) and the frequency and duration of prescribed exercise programmes for people with axSpA. Two systematic reviews of physiotherapy and exercise in axSpA reported that the frequency and duration of exercise programmes ranged from once per week for 9 months to five times per week for 3 weeks [6, 7]. A consensus meeting on recommendations for exercise and physical activity for people with axSpA concluded that the recommended dosage of exercise for benefits in pain, mobility, disease activity and function is still unknown [8].

Adherence to an exercise programme is central to its success. Adherence is defined as the extent to which a person’s behaviour corresponds with the recommendations from a health care professional and is a primary determinant of the efficacy of an intervention (World Health Organisation 2003). Since adherence is a behaviour, measuring this is difficult. A systematic literature review investigated rates of adherence to prescribed exercise programmes in spondyloarthritis and found adherence was poorly reported within nine papers and suggested participants do not fully adhere to prescribed exercise [9]. The reasons for lack of adherence can be multi-factorial, such as socioeconomic status, health condition, intervention-related factors and personal circumstance [10]. Although exercise programmes which are long term and high frequency may improve outcomes for people with axSpA, adhering to prescribed exercise is challenging [8, 11].

Given that internet use is now ubiquitous for most people [12], online programmes offer a potential alternative to face-to-face exercise sessions whilst still allowing the therapist to remotely supervise and review progress. In addition, evidence-based behavioural change techniques such as goal setting and feedback can be incorporated to improve long-term adherence to exercise [13]. Our research group developed a platform for delivering individualised online physiotherapy for chronic conditions (www.giraffehealth.com), which has shown promising results in terms of feasibility and effectiveness for multiple sclerosis and spinal cord injury [14–17].

The aims of this prospective, interventional, cohort study were to assess adherence to a 12-month, individualised, online physiotherapy programme for people with axSpA who were not regular exercisers; to investigate the effects of the intervention on pain, disease activity, spinal mobility, exercise capacity, physical activity level, quality of life, work impairment, motivation and attitude to exercise and functional activity and to determine whether there was an association between the level of adherence to the programme and these outcomes. In addition, participants’ views of the intervention were explored with special consideration of factors affecting adherence.

## Methods

### Participants and ethical approval

The detailed protocol for this study has been previously published [18]. In summary, participants were recruited from rheumatology out-patient clinics in a single, large health board (NHS Greater Glasgow and Clyde) in Scotland, United Kingdom. Study participants were required to have been diagnosed with axSpA for at least 1 year and have access to the internet. Patients were not eligible if they regularly exercised (defined as exercising three or more times per week), had a joint replacement within the last 6 months, had other significant comorbidities for which exercise is contra-indicated or were taking part in another clinical trial. The study was reviewed and approved by the West of Scotland Research Ethics Committee (Ref: 15/WS/0229) and registered on ClinicalTrials.gov (ref: NCT02666313).

### Intervention

Written informed consent was obtained from each participant. A specialist rheumatology physiotherapist assessed all participants, agreed exercise goals with the participants and prescribed an individualised exercise programme via the online exercise platform (Giraffe Healthcare CIC, Glasgow, UK) which included demonstration videos of each exercise, an online exercise diary and axSpA-specific advice and education. Participants were advised to complete their exercise programme three times per week, plus exercises of their choice (e.g. walking, swimming, sport) twice per week for 30 min over 12 months. Participants were asked to record their completed exercises for each session in the online exercise diary. The physiotherapist reviewed the participants’ online exercise diaries every 2 weeks and adapted their exercise programme as clinically indicated. For the first 2 weeks, participants received weekly phone calls from the physiotherapist to check on progress and answer any questions; thereafter, participants could contact the physiotherapist by phone if required but were not regularly contacted, apart from for their study visits.

### Outcome measures

Outcomes were measured by an independent assessor at baseline, 6 months and 12 months (end of intervention). The primary outcome measure was adherence to the online exercise programme, taken from the online exercise diary. This was calculated over 52 weeks (percentage of completed sessions, out of a maximum of 260 exercise sessions; 52 weeks at 5 times per week), and for each of 13 4-week periods. Good adherence was defined as ≥ 60%, i.e. an average of three or more sessions per week. Secondary outcome measures were mainly patient-reported measures and are described in full in Paul et al. [18]. In summary, the measures were the 6-min walk test (6MWT), Bath Ankylosing Spondylitis Functional Index (BASFI), Bath Ankylosing Spondylitis Disease Activity Index (BASDAI), Bath Ankylosing Spondylitis Metrology Index (BASMI), AS Quality of Life (ASQoL), EQ5D, Work, Productivity and Activity Impairment in AS (WPAI), Exercise Attitude Questionnaire (EAQ) and the Exercise Motivations Inventory-2 (EMI-2). In addition, physical activity (steps/day, walking time and sitting/lying time) was assessed over a 7-day period using the activPAL tri-axial accelerometer worn on the front of the mid-thigh (PAL Technologies, Glasgow, UK) [19, 20].

### Telephone interviews

Semi-structured telephone interviews were undertaken with ten participants between the 6- and 12-month assessment points. Participants were purposely selected based on their adherence (to capture a range of adherence levels). The interviews explored participants’ experience of undertaking exercise generally, factors affecting adherence to the intervention and feedback on the intervention.

### Sample size

The sample size calculation was based on two-third of participants (65%) adhering to the programme which was defined as completing an average of 3 exercise sessions per week in each 4-week period, if 50 participants were recruited to the study, then a 95% confidence interval would have a width of ± 13.2%.

### Data analysis

Participant demographic and outcome measures were summarised by mean and standard deviation, number and percentage as appropriate. Adherence was calculated for each 4-week period for each participant as the percentage of exercise sessions completed, summarised by mean, standard deviation and 95% confidence interval (CI). Outcome measures at baseline, 6 and 12 months were reported by mean and standard deviation. Change in outcome measures from baseline at 6 and 12 months was reported as mean and 95% CI, and assessed using paired *t *tests. The association between adherence and age, duration of disease, 6MWT, BASFI, BASDAI BASMI, ASQOL and physical activity at baseline was assessed using Pearson correlation coefficient and Spearman’s Rho correlation, as appropriate. Correlations of  ≥ 0.30,  ≥ 0.50 and  ≥ 0.70 were considered small, moderate and large, respectively [21]. Telephone interviews were audio recorded, transcribed and analysed using thematic analysis where emerging themes and subthemes were identified and agreed between two independent researchers (MM and LP).

## Results

### Participants

**Fig. 1:**
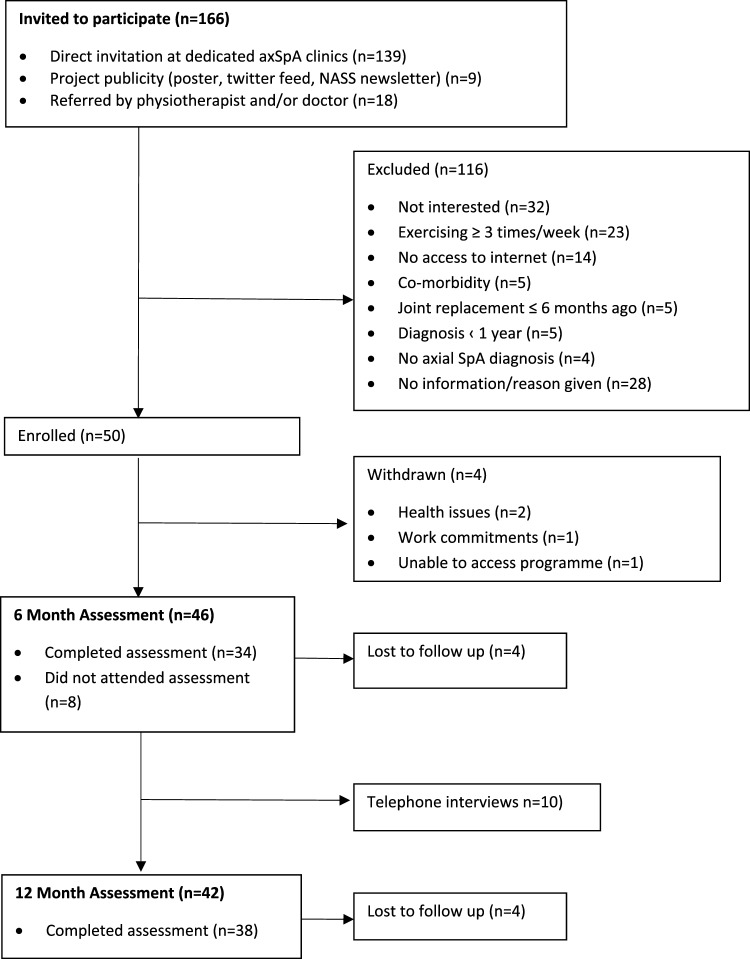
Consort diagram of participants journey through the cohort trial. *axSpA* axial spondyloarthritis, *n* number, *NASS* National Axial Spondyloarthritis Society

Fifty participants were recruited (twenty-three male (46%) and twenty-seven female (54%)) with a mean age of 50 ± 12 years and a mean axSpA disease duration of 16 ± 12 years (Table 1). Four participants withdrew from the study prior to their 6-month assessment due to health issues (*n* = 2), work commitments (*n* = 1) and being unable to access their programme (*n* = 1) (Fig. 1). Four participants were lost to follow-up at 6- and 12-month assessment points.

**Table 1:** Baseline participant characteristics (*n* = 50)

	*n* (%)	Mean ± SD (range)
Demographics		
Age (years)		50 ± 11.7
Gender (M:F)	23:27 (46:54)	
Disease duration since diagnosis (years)		16.2 ± 11.9 (1–45)
Weight (kg)		76.9 ± 18.2 (52–120)
BMI (kg/m^2^)		27.2 ± 5.6 (17–42)
Type of axSpA*		
AS	48 (96%)	
nr-axSpA	2 (4%)	
*n* of self-reported co-morbidities		
0	21 (42%)	
1	18 (36%)	
2	8 (16%)	
3	3 (6%)	
Work status		
Paid employment	34 (68%)	
Retired/medically retired	10 (20%)	
Unemployed	3 (6%)	
Off work	2 (4%)	
Student	1 (2%)	
Mobility		
Mobility with aid (stick)	4 (8%)	
No aid required	46 (92%)	
Current treatments		
TNFi	25 (50%)	
NSAIDs	30 (60%)	
Analgesics	22 (44%)	
Currently attending physio/exercise class	4 (8%)	

*AS* ankylosing spondylitis, *axSpA* axial spondyloarthritis, *BMI* body mass index, *F* female, *M* male, *n* number, *nr-axSpA* non-radiographic axial spondyloarthritis, *NSAIDs* non-steroidal anti-inflammatory drugs, *SD* standard deviation, *TNFi* tumour necrosis factor inhibitor

*Based on consultant diagnosis in medical records

### Adherence

**Fig. 2:**
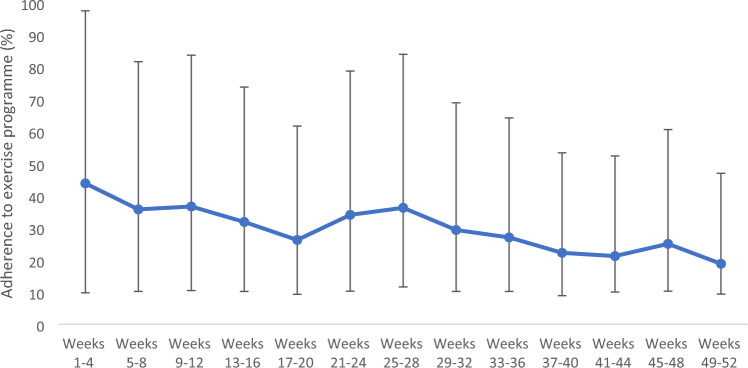
Adherence to exercise programme

Seven participants (14%) had no exercise sessions recorded at any time. Twelve participants (24%) achieved good adherence (≥ 60%) over the 12-month period. Mean adherence was highest during the first 4-week period, at 44% (± 30%) with a 95% confidence interval (CI) of 34% to 54%. Adherence generally decreased over time, with adherence in the last 4-week period at 19% (± 30%), 95% CI 9% to 28%. Temporary increases in adherence were observed around the times when 6- and 12-month study visits were scheduled (Fig. 2).

### Effect of the intervention

Compared to baseline, there were significant improvements in the mean BASDAI, BASMI, 6MWT, AsQoL and EQ5D-VAS at both 6 and 12 months indicating improvements in exercise capacity, disease activity, spinal mobility, quality of life and self-rated health status (Table 2). There were improvements at 6 months only in EQ5D index (quality of life) and the WPAI activity impairment subscale. There were no significant changes in BASFI, physical activity (including steps/day, walking time or sitting/lying time), EAQ and EMI2 (Table 2).

**Table 2:** Participant outcomes at baseline, 6 months, and 12 months

Outcomes			Mean difference from baseline	*p* value
	*N*	Mean (SD)	Mean (95% CI)	
BASDAI (0–10)				
Baseline	50	4.4 (2.5)		
6 months	34	3.5 (2.0)	−0.9 (−1.7, −0.1)	**0.02**
12 months	34	3.4 (2.5)	−1.1 (−1.8, −0.3)	**0.01**
BASFI (0–10)				
Baseline	50	4.1 (2.6)		
6 months	32	3.8 (2.6)	−0.3 (−0.8, 0.3)	0.31
12 months	35	3.6 (2.9)	−0.5 (−1.0, 0.1)	0.10
BASMI (0–10)				
Baseline	50	3.7 (1.9)		
6 months	33	3.2 (1.9)	−0.5 (−0.8, −0.2)	**≤ 0.001**
12 months	33	3.3 (2.0)	−0.4 (−0.7, −0.1)	**0.01**
6-min walk test (m)				
Baseline	50	406.5 (112.2)		
6 months	32	444.3 (109.9)	27.6 (4.6, 50.7)	**0.02**
12 months	34	454.3 (104.5)	43.7 (17.1, 70.2)	** ≤ 0.001**
Steps taken (steps/day)				
Baseline	50	7,784 (3,148)		
6 months	24	7,190 (2,171)	−594 (−1,532, 343)	0.20
12 months	24	6,289 (1,998)	−555 (−1,501, 390)	0.24
Walking time (min/day)				
Baseline	50	93.2 (41.5)		
6 months	24	92.38 (26.86)	−7.40 (−19.47, 4.67)	0.22
12 months	24	83.12 (24.54)	−7.28 (−20.33, 5.77)	0.26
Sitting/lying time (h/day)				
Baseline	50	10.8 (2.3)		
6 months	24	10.28 (2.07)	0.15 (−1.05, 1.35)	0.80
12 months	24	10.39 (2.3)	0.16 (−0.73, 1.05)	0.71
ASQoL (0–10)				
Baseline	50	8.6 (5.6)		
6 months	33	6.7 (5.0)	−1.9 (−3.2, −0.6)	**0.01**
12 months	35	5.9 (5.3)	−2.5 (−4.0, −1.0)	** ≤ 0.001**
EQ5D index (−1 −1)				
Baseline	50	0.7 (0.3)		
6 months	32	0.8 (0.2)	0.1 (0.0, 0.2)	** ≤ 0.001**
12 months	35	0.8 (0.2)	0.1 (0.0, 0.1)	0.07
EQ5D VAS (0–100)				
Baseline	50	66.4 (16.4)		
6 months	32	72.4 (16.0)	6.0 (0.2, 11.8)	**0.04**
12 months	35	71.4 (18.6)	6.6 (1.3, 11.9)	**0.02**
WPAI (activity impairment) (0–100)				
Baseline	50	40 [10 – 70]*		
6 months	32	30 [0 – 55]*	–	**0.01**
12 months	34	30 [10 – 60]*	–	0.15

*ASQoL* Ankylosing Spondylitis Quality of Life, *BASDAI* Bath Ankylosing Spondylitis Disease Activity Index, *BASFI* Bath Ankylosing Spondylitis Functional Index, *BASMI* Bath Ankylosing Spondylitis Mobility Index, *CI* confidence interval, *hrs* hours, *m* metres, *mins* minutes, *N* number, *SD* standard deviation, *VAS* visual analogue scale, *WPAI* Work, Productivity and Activity Impairment in AS

*Indicates median and interquartile range value

*p* values in bold indicate statistical significance

There were no significant associations (as determined by Pearson’s and Spearman’s Rho correlation coefficients as appropriate) between average level of adherence and the following variables; age, BMI, disease duration, BASMI, BASFI, BASDAI, EQ5D index, EQ5D-VAS, 6MWT, ASQoL, physical activity (steps taken/day, walking time or sitting/lying time), EAQ and WPAI at baseline (Table 3).

**Table 3:** Associations between average adherence to exercise and baseline demographics and outcomes

Category	Variable	Sessions completed	
Demographics	Age (*n* = 49)	*r*	0.17
		*p*	0.46
	BMI (*n* = 43)	*r*	−0.118
		*p*	0.44
	Disease duration (*n* = 49)	*r*	0.50
		*p*	0.73
Disease	BASMI (*n* = 49)	*r*	−0.17
		*p*	0.24
	BASFI (*n* = 49)	*r*	−0.218
		*p*	0.13
	BASDAI (*n *= 49)	*r*	−0.138
		*p*	0.35
Exercise capacity	6MWT (*n* = 49)	*r*	0.235
		*p*	0.10
Physical activity	Steps taken (*n* = 44)	*r*	r = 0.017
		*p*	0.914
	Walking time (*n* = 44)	*r*	−0.06
		*p*	0.70
Quality of life	ASQoL (*n* = 49)	*r*	−0.28
		*p*	0.05
	EQ5D—pain (*n* = 49)	*r*	−0.14
		*p*	0.34
	EQ5D—mobility (*n* = 49)	*r*	−0.106
		*p*	0.47
	EQ5D—self-care (*n* = 49)	*r*	−0.66
		*p*	0.65
	EQ5D—usual activities (*n* = 49)	*r*	−0.263
		*p*	0.07
	EQ5D—anxiety/depression (*n* = 49)	*r*	−0.125
		*p*	0.39
	EQ5D—health score (*n* = 49)	*r*	0.20
		*p*	0.20
Attitude to exercise	EAQ (*n* = 49)	*r*	0.2
		*p*	0.20
Work capacity	WPAI (*n* = 48)	*r*	−0.227
		*p*	0.12

*ASQoL* Ankylosing Spondylitis Quality of Life, *BASDAI* Bath Ankylosing Spondylitis Disease Activity Index, *BASFI* Bath Ankylosing Spondylitis Functional Index, *BASMI* Bath Ankylosing Spondylitis Mobility Index, *BMI* body mass index, *EAQ* Exercise Attitude Questionnaire, *N* number, *WPAI* Work, Productivity and Activity Impairment in AS

### Adverse events

There were 19 adverse events recorded during the study, 4 of which were non-related, serious adverse events: cancer diagnosis (*n* = 2), fractured humerus (*n* = 1) and hospital stay due to headache (*n* = 1). Of the remaining adverse events, eight were regarded as related or possibly related to the study intervention or procedures: skin reaction to Tegaderm waterproof dressing used for activity monitor (*n* = 3); axSpA disease flare or increased localised musculoskeletal pain within the first 2 weeks of commencing the study (*n* = 5).

### Participant views

Telephone interviews were conducted with ten participants, five males and five females (20% of the total sample). The participants interviewed were aged between 47 and 79 years, with adherence of between 0 and 69%. The findings of the interviews generated 4 themes and 13 subthemes; the 4 themes were Beliefs and Experiences of Exercise, Factors Positively Affecting Adherence, Factors Negatively Affecting Adherence and Aspects of the Study (Supplementary Table S1).

### Theme 1: beliefs and experience of exercise

There were four subthemes under Beliefs and Experience of Exercise.

### Previous exercise programmes from a health care professional

Participants generally reported that they had been given exercises previously, usually paper based; however, they would forget to do the exercises.

“I was given a sheet of paper of exercises and looked through them once at the hospital and em… I kept forgetting about them and every so often I’d remember and start doing them again for maybe a week or two or a bit longer and I would fall by the way-side” (Participant 24, 45% adherence)

### NASS groups

Participants had mixed views about the National Axial Spondyloarthritis Society (NASS) exercise group classes, with some participants having attended them regularly for many years and others who had decided not to attend due to factors such as location and timing.

“When I did go to the NASS group up at [hospital] by the time I got there driving through the busy part of [city], and then that was tiring after work then by the time I came to drive home I just wanted to sleep…” (Participant 24, 45% adherence)

“I’ve been going to the class a couple of years now.… lots of stretching… lots of aerobic type things” (Participant 44, 8% adherence)

### General exercise

Participants reported trying different forms of exercise with varying degrees of success.

“I did try swimming but I found that the water was too cold and did do a bit at [name] gym for a while but it was expensive, I managed a bit more there but I couldn’t afford to keep it going” (Participant 24, 45% adherence)

“Not until I took up golf… I think it had actually solidified by that time” (Participant 1, 63% adherence)

### Exercise beneficial for condition

Perhaps reflecting that participants had signed up to take part in an exercise study, all agreed that exercise was beneficial for their condition.

“I think the benefits [of exercise] are everything actually. Mm… everything works better … so it makes me feel more like I used to… I think it makes you a happier person” (Participant 1, 63% adherence)

### Comparison with family members

Some participants reported family members, including siblings, also had axSpA and compared themselves and their exercise behaviours to those family members, reinforcing the positive effects of exercise.

“I think it’s [exercise] essential. I’ve got two brothers who have got it. One brother never did exercise. He’s now all bent, can’t stand up straight his joints have all kind of seized up and he can never raise his head again. And my other brother has done a huge amount of exercise … and he, yeah he has flare ups and things like that but like me he knows that if we don’t keep going with it… we could end up like my oldest brother” (Participant 24, 45% adherence)

### Theme 2: factors positively affecting adherence

There were three sub-themes under Factors Positively Affecting Adherence to the intervention.

### Feel better/keep independent

Participants reported that the intervention helped them feel better, in some instances in a similar way to taking medication, and this positive effect supported adherence.

“I don’t want to be stiff and now I’m much more supple, I’m enjoying it and I don’t want to take pain killers if I am sore...obviously exercise is simple, it’s like a tablet isn’t it? If you take it, you aren’t going to be sore. And now I know that if I do get a flare up and I’m sore, maybe a bit of exercise can be the answer” (Participant 2, 44% adherence)

### Getting into a routine

Getting into a routine with exercise was suggested by many participants to positively affect adherence.

“I know that when it [the study] stops I’ll be more inclined to carry on as well because…it’s a mindset thing… I’ve got a better routine now than I’ve ever had” (Participant 24, 45% adherence)

### Support from others

Participants provided many instances where family members or others encouraged participants to do their exercise programme, often doing it with them.

“I could look at my diary and discover like ‘ok I’ll do that day and that day’ but my wife is very good and she forces me into doing it… so we both done it together… its good someone else doing it with you because they give you encouragement” (Participant 2, 44% adherence)

### Theme 3: factors negatively affecting adherence

There were four sub-themes under Factors Negatively Affecting Adherence.

### Symptoms

There were two opposing views of the effect of the symptoms of axSpA on adherence to exercise. Some participants reported that symptoms, such as pain and fatigue, prevented them from doing their exercise programme.

“I had an off day where my hip was sore or my shoulder was inflamed and I found it hard. Also, my neck flared up and even though they say ‘no it helps’ it was just that I wanted to just lie down and sleep to get over it. It’s the fatigue that gets you because you’re tired and when you do exercises you can actually make bits sorer and it can last for days” (Participant 36, 0% adherence)

Conversely when symptoms were well controlled, some participants did not feel the need to do any exercise.

“because my condition is really quite good now between getting the new hips and the good medication, I just don’t feel a great need for it, but the big thing is that if my condition deteriorated then I would need to look at a web-based thing [online exercise programme]” (Participant 44, 8% adherence)

### Life events

Adhering to exercise was adversely affected by events, anticipated or unanticipated, in participants’ lives.

“A lot happened with my aunt being unwell and then we had a holiday and after that it just got on top of me and I didn’t get it done… and it wasn’t because I didn’t want to do it ... other things in life took over” (Participant 2, 44% adherence)

### Lack of motivation

Lack of motivation stemmed from the monotony of the exercise programme or the time taken to complete the programme. Whist for others, it was a feeling of laziness.

“I’ve great intentions that never really materialise” (Participant 44, 8% adherence)

A specific issue raised was that some participants preferred to exercise in a class and were not motivated to exercise alone at home.

“If I had been in a group... that might have helped but it was lonely” (Participant 36, 0% adherence)

### Theme 4: aspects of the study

There were three sub-themes under Aspects of the Study.

### Benefits of taking part in the study

Participants reported a range of benefits of taking part in the study including reducing symptoms, losing weight and generally feeling better.

“It gets you into the habit and you feel good you feel quite proud and between that and the hip operations I’ve lost about 18kg… I have just reached the stage where I can kneel now. It’s been a long time since I’ve been able to manage that… and as I say I’m hardly using any medicine. In fact, that in itself probably makes you feel better” (Participant 1, 63% adherence).

### Positive features of the online physiotherapy programme

There were a number of positive areas of feedback in relation to the online platform and mode of delivery, such as the exercise videos, exercise diary, and the remote monitoring by the therapist.

“A physio gave me some sheets to study and I found it difficult because I didn’t know if I was doing it properly and eh, let’s be honest, you don’t always do it! So for me the web-based was very good because you could actually watch people doing it, so you could follow and know how to do it... it was quite good because I had my diary that I could put down if there were any issues and [the therapist] would follow it up or change it [exercise programme] so that was good” (Participant 2, 44% adherence)

Contrary to the previous point, some participants raised that they did not like group exercise but that exercise at home was more convenient.

“Oh I’m happy with that [exercising at home] I wouldn’t dream of exercising in a group that’s not my character” (Participant 25, 12% adherence)

### Negative features of the online physiotherapy programme

Participants also highlighted the negative aspects of the online physiotherapy platform, including the inconvenience or non-completion of their exercise diary, internet connectivity issues and lack of variety of the exercise programme given.

“So a few times, or quite a lot of times I’ve not put it in [added to the exercise diary] but I’ve done all the exercises… it’s a bit frustrating” (Participant 24, 45% adherence)

“The only problem is that if you don’t have internet access that can be an issue” (Participant 2, 44% adherence)

## Discussion

This prospective, interventional cohort study demonstrated that adherence to an online physiotherapy-led exercise programme, five times per week over a 12-month period, declined from 44% in the first 4 weeks, to 19% in the final 4 weeks of the year-long programme. Initially, 24% of participants demonstrated good adherence, defined as completing at least three sessions per week, but this declined to 7.5% by the end of the year. Temporary increases in adherence were observed around the times when study visits were scheduled.

Adherence rates of between 19 and 44% could be considered to be low; however, there is no agreement on what constitutes acceptable or low adherence [22, 23]. Furthermore, with no standard method of measuring adherence, and adherence rates reported to highly heterogeneous exercise programmes, in terms of length, duration and type of exercise intervention, direct meaningful comparison of adherence rates is almost impossible. The current study required participants to exercise five times per week for 12 months whereas the majority of exercise studies prescribe exercise twice per week over a short duration such as 12 weeks, exercising in the short term is likely less challenging than maintaining an exercise programme over the longer term [6, 7]. Furthermore, the current study specifically targeted a population who were not regularly exercising prior to recruitment into the study. Therefore, exercising five times per week was likely too much of a change in exercise habits; which may account for 14% of participants who did not commence their exercise programme. A lower intensity may have been more achievable. In addition, several participants reported doing the exercises without recording this in their exercise diary; therefore, adherence in the current study is likely under-reported. This study found that exercise adherence reduced over the course of the intervention period, a reduction in exercise adherence over time is echoed in previous research. For instance, Pisters et al. [24] reported 75% adherence at 13 weeks and 59% adherence at 65 weeks following an individually tailored home-based exercise programmes for people with osteoarthritis. Similar findings have been found in online delivery of exercise over 8 weeks and 6 months in other long-term conditions such as type 2 diabetes and multiple sclerosis [16, 25–27]. Furthermore, a recently published cross-sectional study reported with 79% of participants with AxSpA had poor adherence to exercise, measured using total score on the Exercise Attitude Questionnaire [28].

The participant interviews gave context to the adherence data, with participants reporting that factors such as getting into a routine and social support increased their adherence, whereas other life events and lack of motivation reduced their adherence. Interestingly, there were different opinions in terms of the effect of the symptoms of axSpA on adherence. Some felt increased symptoms, such as pain and fatigue, reduced adherence and when they felt better, their adherence improved. In contrast, others reported when they had few symptoms, they did not feel they needed to exercise and their adherence reduced. There were also differences of opinion in terms of the mode of exercise; some liked the convenience of exercising at home and would not have liked to exercise in a group setting, whereas others would have preferred a supervised group setting.

AxSpA flare ups were reported by five participants upon commencing the exercise programme, suggesting these were potentially related to the study intervention. These may have resulted due to the sudden increase in exercise frequency and/or intensity, given that the participants were not regular exercisers prior to this. Disease flares are a common occurrence as part of the disease course, it has been reported that approximately 30% of people with axSpA report a flare on any given week [2]. The reported flares may be part of the natural disease course recorded due to frequent contact with the study team as well as due to increased activity as part of starting a new exercise programme in previously inactive people. Therefore, as a precaution, the intensity of the exercise programme was started more gradually for subsequent participants with no further axSpA flare ups reported.

There were significant improvements in the BASDAI, BASMI, 6MWT, AsQoL and EQ5D-VAS at both 6 and 12 months, indicating improvements in exercise capacity, disease activity, spinal mobility, quality of life and self-reported health status over the study period. There were improvements at 6 months only in EQ5D index and the WPAI activity impairment subscale. There were no significant improvements in remaining outcome measures and no associations between level of adherence and participant demographics or baseline variables. Improvements at 12 months met the Minimal Clinical Important Difference (MCID) for the BASDAI (1.1) and almost met the MCID for the BASFI (0.6) [29] Mean improvements in the 6MWT were below the MCID for the 6MWT in people with neck and back musculoskeletal pain (60 m) [30]. This study did not include a control arm; therefore, whilst the results are positive, they should be interpreted with caution. However, the improvements in clinical outcomes in the current trial agree with findings from two previous systematic reviews which indicated that online delivered exercise programmes have the potential to be as effective as traditional methods of exercise prescription in people with axSpA [6, 7]. Digital health has the added benefit of allowing remote delivery of exercise and greater flexibility for asynchronous monitoring, particularly for those who have family/work commitments, require significant travel to attend supervised or group exercise [31, 32], although internet issues were reported as an issue in a small number of participants. Furthermore, use of digital health to reduce “health miles” contributes to the climate change agenda [33].

### Limitations

This study had a number of limitations. It is possible that the study is subject to selection bias as only participants interested in this model of physiotherapy exercise may have agreed to take part. However, the inclusion criteria meant that only people who were not regularly exercising were eligible for the study, as such they may have been in the contemplation phase of the transtheoretical model of behaviour change and may not have progressed to be able to maintain their exercise regime. Adherence was measured using self-report electronic diaries that were not recorded automatically and could not be completed retrospectively. As such, adherence to the programme was likely under-reported. Whilst the aim of the current study was to explore the adherence to exercise in the long-term, no control group was recruited; therefore, changes in clinical outcomes cannot be assumed to be due to the intervention and may have occurred anyway or been as a result of other treatment or lifestyle changes. It is difficult to compare adherence rates from the current study to previous research, as previous exercise programmes were generally of lower intensity and shorter in duration. Finally, in the literature, adherence has been measured using different methods with no consensus on how to measure or rate adherence.

## Conclusion

Adherence to an online exercise programme of five times per week over 52 weeks varied from 44%, decreasing to 19% in the final 4-week period and may have been under-reported. This study observed significant improvements in disease activity, spinal mobility, exercise capacity, quality of life and self-reported health status in people with axSpA who were not regular exercisers. Participants reported establishing a routine and social support contributed to their adherence whilst life events and lack of motivation reduced adherence. There were differences in opinion on the effect of symptoms on adherence. Online exercise programmes may benefit people with axSpA; however, strategies to improve adherence are required.

### Supplementary Information

Below is the link to the electronic supplementary material.

Supplementary file1 (DOCX 33 KB)

## Data Availability

Data available on request.
