# Supplementary material for: Online physiotherapy for people with axial spondyloarthritis: quantitative and qualitative data from a cohort study
Source: Rheumatol Int. 2023 Sep 21;44(1):145–56. doi: 10.1007/s00296-023-05456-6 (PMC10766789; doi:10.1007/s00296-023-05456-6)
Supplement: Supplementary file 1 — Supplementary file1 (DOCX 33 KB) [file 296_2023_5456_MOESM1_ESM.docx]

**Title:** Online Physiotherapy for people with axial spondyloarthritis – quantitative and qualitative data from a cohort study

Paul L^1^ McDonald MT^1^, McConnachie A^2^, Siebert S^3^, Coulter EH^1^

**Affiliations:** ^1^School of Health and Life Sciences, Glasgow Caledonian University, Glasgow, UK; ^2^Robertson Centre for Biostatistics, School of Health and Wellbeing, University of Glasgow, Glasgow, UK; ^3^School of Infection and Immunity, University of Glasgow, Glasgow, UK

**Corresponding author**

Dr Elaine Coulter

[Elaine.Coulter@gcu.ac.uk](mailto:Elaine.Coulter@gcu.ac.uk)

**Supplementary Table S_1_.** Findings from semi-structured interviews.

| Beliefs and experience of exercise | Previous exercises from a health care professional | well I was given some by the physio when I first got all the details of being diagnosed …. and I was given things to do but em I guess there was no follow up I suppose….every now and again the physio would take some measurements to give me a score (P25)  I was given a sheet of paper of exercises and looked through them once at the hospital and em I kept forgetting about them and every so often I’d remember and start doing them again for maybe a week or two or a bit longer and I would fall by the way side (P24)  well I suppose what I was given in the past was one go at it or being shown it and never really attempting some of it and just given some line drawings (P24)  If anybody offered me hydrotherapy I would be there but other than that (P24)  I was advised by [consultant] and he gave me specific exercises, stretching, rocking the pelvis when you get up in the morning so the base of your spine doesn’t fuse (P36)…  years ago but mostly it was just a bit of paper and it just had somebody touching their toes, doing apart from [consultant] he showed me he did the exercises in front of me (P36) |
| --- | --- | --- |
|  | NASS Group | The AS class and stretches .. I downloaded the [NASS] app (P17)  I joined the NASS group and I got a disk from them of exercises but I don’t always remember to do it(P25)  When I did go to the NASS group up at [hospital] by the time I got there driving through the busy part of [city], and then that was tiring after work the by the time I came to drive home I just wanted to sleep… (P24)  also they [NASS classes] were like for an hour, hour and a half and I wasn’t sure of the area so I wasn’t even sure how to get there (P36)  I’ve been going to the class a couple of years now.… lots of stretching… lots of aerobic type things (P44) |
|  | General Exercise | I used to go to, that woman on the tv she used to have classes, it was diet classes plus she had exercises. I found it was a bit too strict for me and I have looked for exercise classes literally for over 60s but you cant get them anywhere. (P36)  I did try swimming but I found that the water was too cold and did do a bit at David llyod gym for a while and it was expensive, I managed a bit more there but I couldn’t afford to keep it going. (P24)  and I used to do yoga which I found was good apart from I had problems getting my back up off the floor…. I did karate but it was too jerky , far too, well I’m not that bendy. (P36)  No [previous exercises for AS].. Not until I took up golf… I think it had actually solidified by that time (P1) |
|  | Exercise beneficial for condition | it keeps you independent (P25)  it enables me to finish a job or knowing that I can tackle a job. I know that sitting in an armchair for long is no good (P25)  I think the benefits are everything actually. Em everything works better … so it makes me feel more like I used too….I think it makes you a happier person (P1)  I think you get more flexible... I always say to myself if did exercises back in the day it wouldn’t be so bad, (P17) |
|  | Comparison with family members | Exercise is very important. My mother had it ….and was told don’t move your neck, she was given a collar and told if you’re in pain just sit, don’t do any exercise don’t move. And she literally did that and she ended up with a curved spine and her tailbone had actually fused. When I started going to the hospital I was told, move, exercise is important (P36)  I think its essential. I’ve got two brothers who have got it. One brother never did exercise. He’s now all bent, cant stand up straight his joints have all kind of seized up and he can never raise his head again. And my other brother has done a huge amount of exercise … and he, yeah he has flare ups and things like that but like me he knows that if we don’t keep going with it… we could end up like my oldest brother (P24). |
| Factors negatively affecting adherence |  | thing if you feel the pain is worse then you don’t do as much as you should (P25)  Theres occasional days when theres so much family stuff going on that I’m just too tired to do them all. I do some of them every day but I don’t do them all every day (P24) being tired (P24)  if im standing in the office waiting for the kettle to boil I should go back and sit down while Im waiting but if theres nobody about il d some of the standing exercises. Some of colleagues know what im doing and just have a laugh at me ,,,,then at night time I sit and do what ive not done (P24)  phases when I was especially busy before xmas and things and more tired and I wasn’t able to do as much and just wee phases when Ive been having a flare as well (P24)  I struggle with that and actually I like the group’s exercise… I like activity and music and just being around other people when I do it… I’m not very good at motivating myself to do it at the house (P44).  A lot of people maybe have the inclination to do it. I’m afraid I’m just a bit lazy when it comes to things like that… I’ve great intentions that never really materialise (P44)  because my condition is really quite good now between getting the new hips and the good medication I just don’t feel a great need for it, going to the class but the big thing is that if my condition deteriorated then I would need to look at a web based thing where I have to do it in the house (P44)  For me obviously, there is no substitute for going to a class an exercise class which was lead by a physio…Which has music and stuff…Yeah definitely I like and just being around people that have got the same thing, you can relax (P44).  Over the year… I think… I think I attended more at the beginning of the course and it just kind of tail off. I was ashamed to say (P44) *Interesting use of the word ashamed*  I do …partly because of my hip, getting my hip replacements and making a commitment to go to the class after that because I really appreciated having my mobility back and I really felt the loss of it quite keenly so I’ve been going solidly since (P44)  When you’re retired you have a much more flexible programme. (P1)  after the operation I was on these painkiller and I was taking the maximum, two every four hours. Now I take one just before my morning walk, because if the pain starts up then your inclined not to walk as much as you should. (P1)  unfortunately a lot was happening last year and its very easy to drop by the way side and I did do that, unfortunately other things in life took over.(P2)  well started off very good ….And we looked forward to doing it and I think it was about November that we fell away. A lot happened with my aunt being unwell and then we had a holiday and after that it just got on top of me and I didn’t get it done. Right and it wasn’t because I didn’t want to do it (P2)  I think it was just up to me… probably emailed me and telt me to get off my arse. [emailed]monthly to say look we’ve been watching you you’re not doing enough, that’s what I do need (P17)  just everyday things, well the kids popping in- they’re adults – but people popping in, feeling tired, having a nap, realising you had to make dinner, or getting up in the morning and thinking Im going to do it right now and getting distracted with making breakfast. With the diabetes I had to eat and then once you eat and think exercise, youre tired because you’ve ate ad glucose levels have shot up. if I had been in a group .. that might have helped but it was lonely (P36)  when I had an off day where my hip was sore or my shoulder was inflamed and I found it hard. Also my neck flared up and even though they say no it helps it it was just that I wanted to just lie down and sleep to get over it It’s the fatigue that gets you because you’re tired and also when you do exercises you can actually make bits sorer and it can last for days. (P36)  I found the monotony of going on and going through exercises hard ..yeah the logging on was kind of distracting so it was…. its so time consuming (P36) |
| Factors positively affecting adherence | Feel Better/Keep independent | because I find it works…. because it makes me feel better (P25)  well its being 80 and wanting to be independent. I have friends who are so unable to do things that it’s a frightening prospect (P25) so you feel that you, you know, haven’t got many years left on and some of them could be really bad, that frightens me so I feel I need to keep going. (P25)  I don’t want to be stiff. And now I’m much more supple I’m enjoying it and I don’t want to take pain killers if I am sore so obviously exercise is simple, it’s like a tablet isn’t it if you take it you aren’t going to be sore. And now I know that if I do get a flare up and I’m sore, maybe a bit of exercise can be the answer (P2) |
|  | Routine | I know that when it stops I’ll be more inclined to carry on as well because I’m now, it’s a mindset thing but even kind of driving into work in the morning my stiffness is wearing off and I’m starting to do the neck ex a bit as I’m driving into work and then doing them again later on. I’ve got a better routine now than I’ve ever had (p24)  the main thing is to get into a routine and then you actually enjoy it. Look forward to it. In fact you miss it if you don’t do it. Routine is one of the main but also feeling good (P1) |
|  | Family support | .. I think summer time I did a wee bit more as well plus I was going to ____ [holiday] so I thought I better get out and do something .. looking in the mirror sometimes and getting encouraged to do it. My partners on my case so so I better do it (laughs) (P17)  I could look at my diary and discover like ok I’ll do that day and that day but my wife is very good and she forces me into doing it … so we both done it together so she was good at saying you’ve got to do it but eh yeah. You cant fall by the way side, its good someone else doing it with you because they give you encouragement (P2) |
|  | Support from HCP | its good, I need people to see I’m doing it right, I need pushed to do things.. Like when you say to me you want to check it, I mean that’s good (P17) |
| Benefits of taking part |  | It gets you into the habit and you feel good you feel quite proud and between that and the hip operations I’ve lost about 18kg… I have just reached the stage where I can kneel now. Its been a long time since I’ve been able to manage that. I can get down on the floor which I had no chance of doing before. That’s a mixture of losing the big belly and exercising (P1).  And as I say I’m hardly using any medicine. In fact that in its self probably makes you feel better (P1).  oh definitely, Ive stopped being so stiff and I think I actually lost a bit of weight… initially my wife said she got a bit of benefit too so I think yeah the more I got into it I got more supple and felt a lot fitter. Definitely yeah. When I first started I was out of puff but later on in the programme I just got stuck into it and it wasn’t an issue. There was sometimes a bit of groaning and stretching but the way we always found them hard but that’s exercise. I was sore at the start…. I’m not sore anymore (P2)  I’m getting longer with the exercises, I found I could do them for longer and was getting more stamina… the pain of stiffness is less….yes more nimble than I was before. The neck exercises were a good thing because you’re not sure with our necks that tend to fuse, we’re not sure how to move it properly and it was just the knowledge that you could actually do that and I’m allowed to move it that way (P36)  my mobility has maybe got a wee bit better and I think my neck is a wee bit better as well but I think I’ve got slightly more stamina…. A bit less pain when I’m walking I can get a bit further without the pain getting bad (P24) |
| Web-based physio-therapy programme | Positive features of the programme | its good and its certainly made me remember a lot more. I have no criticisms at all and I think its been so far a good thing for me (P24)  it was simple and straight forward…. I thought it was very easy to use and user friendly. It was a nice format, easy to use, I think (P44)  It’s great [exercising at home] You’re more likely to do it (P1).  Oh I’m happy with that [exercising at home] I wouldn’t dream of exercising in a group that’s not my character (P25)  but as far as I’m concerned its perfect doing it in the house. It’s much more convenient and I’m much more likely to do it. …and after a while you look forward to it (P1). … I think its great (P1)  its very good because you can do it in your own time which is good and so obviously do it when you want to. Personally I’d rather do it at home, I wouldn’t want to do it in a group, I don’t really like group activities and I think a lot of people would rather do it. Well its up to the individual, some people like doing group activities, some people might get embarrassed but eh, for me, I’d rather do it on my own (P2)  a physio they gave me some sheets to study and I found it difficult because I didn’t know if I was doing it properly and eh, lets be honest, you don’t always do it! So for me the web-based was very good because you could actually watch people doing it so you could follow and know how to do it..yeah I think for me watching somebody do something is much better than a leaflet (P2)  The good thing is with this programme is that it’s recommended for you, so you telling me what to do for my condition. So I’d hate to do something off youtube or a DVD and discover that I’m doing it wrong or causing more damage (P2)  Much better [than previous exercise programmes], bigger range of exercises so that was good and a follow up which helped. I think for me it was quite good because I had my diary that I could put down if there was any issues and you would follow it up or change it so that was good (P2) |
|  | Negative features of the programme | I didn’t like analysing how I felt in such detail I suppose. It made me more conscious of any extra pain (P25)  So a few times, or quite a lot of times Ive not put it in but Ive done all the exercises… it’s a bit frustrating (P24)  A virtual physio… music, that’d be good (P44)  The only comment I’d make is my progress now cos although I started in December I didn’t catch up with it until April I guess. So the exercises some of them I couldn’t do to begin with to begin with now I feel I could upgrade…. I didn’t realise you read this comments box (P1).  The only problem is that if you don’t have internet access that can be an issue. My problem was that, I’ve got internet in the house but my tablet, I had problems with my tablet so that caused a bit of trouble… cos I’m not good with computers so I struggled initially. . Sometimes it wouldn’t upload and sometimes it would, eh, I couldn’t get volume on it. And then later it was ok (P2)  Maybe mixing up the exercises (P17)  .. individual exercises is not good. You’re watching somebody and then all of a sudden the instruction was over in literally a minute, two minutes at the most. And I think how do I do that?... If it had been just like a DVD, then you could actually go well my hips sore, I can’t do that and just wait ‘til the next one. Also it loosens you up more cos you’re actually in flow, you’re going from one exercise to another, you’re not cooling down and then starting again… exercising at home is terrible (P36)  There’s too many things that can distract you [exercising at home]. oh damn do I have to, and then you’re letting the dog out, going for messages, watching a tv programme, you’re tired, your backs sore your legs are sore. And also you’re by yourself (P36) |
